# Effect of risk factor-tailored autonomy enhancement education in the first-time middle-aged patients undergoing percutaneous coronary intervention: a randomized controlled trial

**DOI:** 10.1186/s12912-023-01654-6

**Published:** 2023-12-18

**Authors:** In Ae Uhm, Seon Young Hwang

**Affiliations:** 1 Inje University Sanggye Paik hospital, Seoul, South Korea; 2 College of Nursing, The Hanyang University, 222 Wangsimniro, Seongdong-gu, Seoul, South Korea

**Keywords:** Coronary artery disease, Middle aged, Risk factors, Self-care

## Abstract

**Purpose:**

It is important for middle-aged patients who have undergone acute treatment for coronary artery disease to voluntarily improve their lifestyle and risk factors based on their recognition as patients with chronic diseases. This study conducted individual education to improve risk factors and tried to verify its effectiveness.

**Methods:**

The hypothesis was verified by applying a randomized controlled pre- and post-test design through random assignment of two groups. Middle-aged (40–64 years) patients who underwent percutaneous coronary intervention for the first time were recruited from a university hospital in Seoul, Korea. For the experimental group, based on the Self-Determination Theory, risk factor- tailored counseling and education were provided individually for one hour based on the education booklet, and telephone counseling was conducted twice for 12 weeks. Differences in autonomous motivation, resilience, self-care compliance and biochemical indicators measured after 12 weeks in the experimental group and the control group were compared. Data were analyzed using SPSS/WIN ver. 22.0.

**Results:**

The autonomous motivation, resilience, and self-care compliance of the experimental group were significantly higher than those of the control group (*p* < .05). Body mass index (*p* = .005) and current smoking rate (*p* < .001) were also significantly decreased in the experimental group but there was no significant difference in other biochemical parameters.

**Conclusion:**

For middle-aged patients with first coronary intervention, risk factor-tailored education emphasizing autonomy by nurses should be provided early after discharge.

**Trial registration:**

This study was retrospectively registered in the Clinical Research Information Service and the identification number is KCT0008698(11/08/2023).

**Supplementary Information:**

The online version contains supplementary material available at 10.1186/s12912-023-01654-6.

## Introduction

Cardiovascular disease is the leading cause of death worldwide and is the second leading cause of death in Korea. Over the past decade, the mortality rate has increased by approximately 16.1%, from 46.9% in 2010 to 63.0% in 2020 [1]. Although the incidence of coronary artery disease (CAD) is high in old age, percutaneous coronary intervention (PCI) procedures are increasing as the number of middle-aged patients aged 40 to 64 has rapidly increased over the past five years [2]. A cohort study that followed 13,000 patients with acute myocardial infarction in Korea for five years found that the success rate of PCI was 99.4%. However, attention has mainly focused on the prognosis after PCI procedures, with major adverse cardiac events, such as restenosis or heart failure occurring after a year in 9.6% of the patients receiving PCI. This increased to 18.8% after two years, with 6.8% of the patients reporting readmissions or death [3].

Typical risk factors for CAD include dyslipidemia, high blood pressure, diabetes, obesity, family history, and smoking, most of which are controllable through lifestyle modifications [4]. Patients who experienced major adverse cardiac events within a year of PCI had a significantly higher prevalence of other chronic diseases, such as hypertension and diabetes [5]. Smoking was a major risk factor for major adverse cardiac events in this period [6]. Studies on the prognostic course of patients who underwent PCI showed that the mortality rate at recurrence was approximately four times higher than that at the onset [7]. Recent advances in treatment technology have led to an increase in the number of PCIs that use the radial artery instead of the femoral artery. This has reduced the absolute stability time after the procedure and freed up activity [8]. This can lead to a lack of recognition of oneself as a chronic disease after acute-phase treatment which can reduce risk factors and the degree of self-care compliance, such as lifestyle improvements and medications. Therefore, it is necessary to develop an educational strategy for first-onset patients to recognize that they are chronically ill at an early stage after PCI and to increase self-motivation to correct risk factors and lifestyle.

In particular, midlife is an important time in the life cycle, when biological aging begins and the incidence of chronic diseases dramatically increases. This can lead to poor health, especially if social, environmental, economic, and psychological factors are not favorable [9]. Middle-aged patients with CAD tend to find treatment procedures such as PCI stressful and urgent. In order to overcome these negative perspectives, patients with cardiovascular disease need resilience, which refers to the belief in positive recovery at the personal level and the power to recover through positive interactions with supportive resources at the relational level [10]. As an optimal strategy to prevent recurrence of CAD, an individual’s autonomous motivation for continuous behavioral change is required along with therapeutic intervention [11]. In the course of follow-up after PCI, the risk of recurrence was 3.9 times higher in the group with low recognition of autonomy support for more than 1 year compared to the group with high recognition [12]. If CAD develops in middle age, since there is still a long life expectancy left, it is necessary to clearly recognize one’s disease and risk factors at the time of onset and actively participate in lifestyle improvement and treatment process based on autonomous motivation. In addition, in a study targeting CAD patients, it was reported that the higher the resilience, the higher the self-care compliance, so it is necessary to increase the resilience [13]. This resilience was found to be improved by increasing self-regulation and interpersonal skills as a result of a 12-week coaching program for middle-aged women [14]. Therefore, it is necessary to strengthen self-management in daily life by increasing the resilience, which is a necessary factor for patients with chronic diseases.

**Fig. 1:**
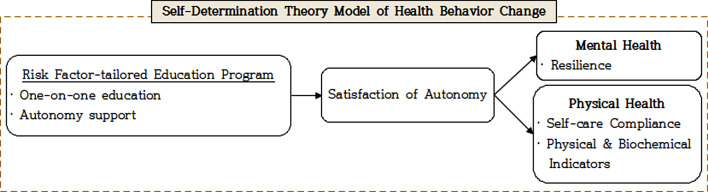
Theoretical framework

The conceptual framework of this study used health behavior change model based on Ryan et al.’s Self-determination theory, which focuses on autonomy to make one’s own choices [15] (Fig. 1). If the patient’s basic psychological need for autonomy is met by providing customized risk factor education that supports the role and autonomy of patients with chronic diseases, nurses are expected to improve the patient’s mental resilience, self-management performance, and physical indicators. Therefore, this study aims to provide counseling education based on interaction between nurses and patients to middle-aged patients who need voluntary improvement on risk factors based on their recognition as patients with chronic diseases after PCI, and to verify its effectiveness.

## Methods

### Study design

This study used a randomized control group pre- and post-test design to confirm the effectiveness of nurse-led individualized education program tailored to risk factors. The research design, application and analysis process of this study was conducted in compliance with the recommendations specified in the CONSORT statement.

### Setting and participants

**Fig. 2:**
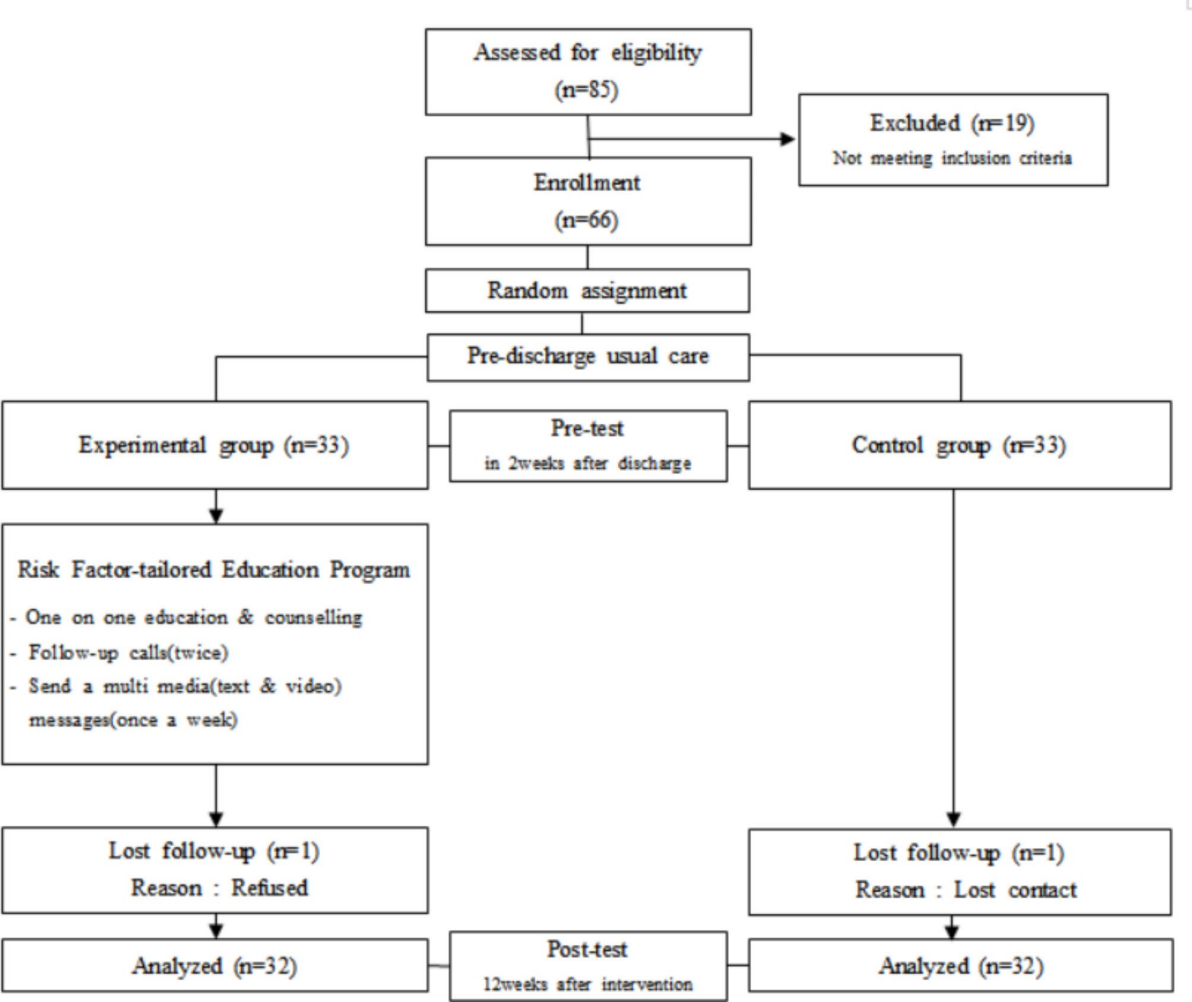
Flow chart of this study participants

Calculation of the appropriate number of subjects required for the study was based on a previous study [16] that verified the effects of disease-related knowledge, health behavior, and quality of life by applying a program using mobile phone apps to patients who received PCI. Using G∗Power program 3.1, the number of samples that fit the t-test with an effect size of 0.80, significance level of 0.05 for the two-tailed test, and power of 80% was at least 26 in each group. The sample comprises first-time middle-aged patients who have undergone PCI after being diagnosed with CAD at the department of cardiology, Seoul, Korea. In total, 85 patients were screened, of which 66 were enrolled as study participants, excluding 19 who did not meet the inclusion criteria. A block randomization method was used to allocate participants into the experimental and control groups using the “rand” function in Microsoft Excel. The block size was 10 and the allocation ratio was 1:1 (Fig. 2). Inclusion criteria were patients aged between 40 and 64 who have received PCI and have CAD for the first time and have at least one of the cardiovascular risk factors [4].

In addition, they were able to communicate, had no difficulty in reading, can use the app using a mobile phone, understand the purpose of this study, and voluntarily gave written consent. Exclusion criteria were those who progressed to heart failure after receiving PCI, those who were diagnosed with mental illness, and those who did not consent to the study.

### Development of an intervention program

We conducted a literature review and a preliminary survey to identify problems in 10 middle-aged patients who received PCI to confirm the need for customized nursing intervention for risk factors with an autonomy support strategy. A booklet was created based on the National Health Information Portal [17] of the Agency for Disease Control and Prevention and the Korean Society of Cardiology’s [18] data related to CAD. It included information on the causes, symptoms, treatments, and risk factor management methods of CAD. Based on the results of a previous risk factor cluster study for patients with CAD [19], learning contents for risk factor management were divided into ‘management of chronic diseases’, ‘obesity and physical inactivity’, and ‘smoking and drinking’. The booklet provided spaces to write down height and weight, body mass index, waist circumference, blood pressure, blood sugar and cholesterol levels. The patients were encouraged to fill in the blanks accordingly. Patients were encouraged to make their own plans to reduce their risk factors. As a tool for autonomy-based action, blood pressure and blood sugar were recorded in the booklets on a daily basis. In addition, mobile applications for each risk factor and a risk factor management log necessary for counseling the patients were included. The intervention application period for the experimental group was decided to be provided for a total of 12 weeks and consisted one face-to-face counseling education (60 min) and two telephone consultations. Also, using social networking services(SNS) we sent one weekly message containing photographs and video materials to continuously discourage priority risk factors. Based on the Self-determination theory, it was decided to use asking questions, advising, assisting, helping with choices, empathizing, and feedback as autonomy support activity strategies when counseling patients.

Lastly, the validity of the patient education program was verified by 10 experts, including two nursing professors, three cardiologists, and five cardiovascular clinical nurses. The content validity index(CVI) of the 15-items validated according to Lynn ’s (1986) evaluation method [20]. A 4-point Likert scale was used to determine whether the educational materials are appropriate for the program goals, whether the educational content is appropriate, whether it is composed of expressions that can be easily understood by the target group, and whether it is easy for the target to carry out self-care. For the CVI evaluation, the researcher met with the expert in person to collect data and receive feedback. The CVI was calculated by adding up the scores of all 10 experts and it was 0.90, confirming content validity. The revised intervention program was finalized by conducting a preliminary survey on three middle-aged outpatients with first-time CAD who underwent PCI, excluding the study subjects.

### Data collection procedure

We recruited participants for the study conducted from May 1, 2021, to October 2, 2021.

All interviews for data collection were conducted by the researcher, the first author, from the experimental and control groups. The participant interview guide, the manual, was developed only for this study and was conducted after obtaining consent after explaining it to the participants according to this guide. Pre-surveys were conducted within two weeks of the first outpatient visit after discharge. The experimental group was provided with a book produced by the researcher. After educating about CAD for 60 min, the researchers conducted counseling education based on an autonomous support strategy for individual risk factors. If any of the three risk factors (‘chronic disease management’, ‘obesity and reduced physical activity’, ‘smoking and drinking’) overlapped, education was conducted on all of the risk factors. The patients were encouraged to choose their preferred application. After the individualized education, a total of two telephone interviews were conducted during the fourth and eighth weeks. In the telephone interview, we consulted whether the plan was implemented well, and if not, what were the barriers (Table 1).

The control group received an individualized education and usual care on post-discharge risk factor management by a nurse for 15 min prior to discharge after PCI.

The post-survey was conducted face-to-face by the researchers during an outpatient visit at the 12th week after the intervention, and biochemical indicators were collected through electronic medical records.

**Table 1:** Composition of risk factor-tailored education program emphasizing patient autonomy

Learning objectives		1. Recognize your illness and risk factors, and set goals for improvement. 2. Recognize the importance of having patient autonomy during illness. 3. Practice self-modification of risk factors and self-care in daily life.		
Week	Topics (Time)	Educational contents	Autonomy-based activities	Autonomy support strategy
1	Establish goals & action plans (1 h)	• Program introduction and participation pledge • Journey as a patient with chronic illness • Reasons for having autonomy to improve risk factors and lifestyle • Recognition of the need to reduce risk factors and treatment of coronary artery disease	• Recognizing your risk factors • Share your thoughts about your illness • Reflect your lifestyle and identify problems • Knowing the age of your blood vessels by numbers (the result of blood tests) • Check your weight and waist circumference • Write down your goals and action plans	Ask Advise Assist Empathize Feedback --------------- One-on-one counseling, education
2 - 11	Risk factor tailored Self-management (use of app)	• Check the degree of goal achievement • Identify and reflect on your own problems • Record through your own risk factor management application • If self-care is not performed, check the cause and suggest alternatives	• Use the application for your risk factors – Hypertension, Diabetes * “MY THERAPY”*, * “Huray Care”* – Obesity, Physical inactivity * “Daily exercise/home training”* * “YAZIO coach”* – Smoking, Heavy drinking * “Smoking cessation assistant,” “Koala”*	Ask Advise Arrange Empathize Feedback -------------- Phone counseling (2 times) & Sending texts & videos weekly
12	Wrap-up (1 h)	• Continuing confidence in risk factor modification and self-care	• Promise to continue practicing self-care in daily life	Emphasize Feedback

### Measurement variables

#### Participants’ characteristics

We collected information on the general characteristics of the participants, including age, sex, marital status, cohabitation type, and education level, immediate family history, and previous diagnosis. To compare risk factors related to the participant’s lifestyle, current smoking, heavy drinking (≥ 8 times/month), and lack of exercise (never, irregular) were collected before and after the intervention,

#### Autonomous Motivation

For the measure of autonomous motivation, the Treatment Self-Regulation Questionnaire modified for patients with diabetes by Williams et al. [21] was used, which was based on the self-regulation instrument of Ryan and Connell [22]. Autonomous motivation was measured with 8 questions from the Treatment Self-Regulation Questionnaire, which was translated into Korean by Park (2015) [23]and verified for validity and reliability through back-translation and application. It was used in this study with permission from the author. Each question was scored on a Likert scale, from 1 (“not at all”) to 7 (“very much”); the higher the score, the higher the tendency to be autonomous and motivated. Cronbach’s α was 0.80 at the time of tool development, and 0.93 in this study.

#### Resilience

For resilience, a total 25-item questionnaire, a resilience measurement tool for cardiovascular disease developed by Shin [24], was used with the permission of the author. It was divided into multiple sub-sections comprising 6 questions about supportive relationships, four on health planning practice, five on control, four on positive attitude, two on recovery beliefs, two on relationships with medical staff, and two on overcoming confidence. Each item was measured using a 5-point Likert scale from 1 to 5 and a total scale range of 25–125; the higher the score, the more resilient an individual is. Cronbach’s α was 0.84 at the time of development and 0.91 in this study.

#### Self-care compliance

For self-care compliance, the self-care behavior tool for medication, diet, exercise, smoking cessation, and follow-up management of patients with coronary artery disease measured by Song [25] was used after obtaining permission from the author. The questionnaire was divided into multiple sections comprising four questions about dosing, 13 about diet, eight about exercise and activity, one about smoking, and four about follow-up management (such as outpatient visits). Each question was measured on a 5-point Likert scale (1 for “not at all,” 2 for “very little,” 3 for “moderate,” 4 for “relatively well,” and 5 for “always doing well”). The total score ranged from 30 to 150; the higher the score, the better the self-care compliance. Song’s study showed a Cronbach’s α = 0.90. In our study Cronbach’s α = 0.89.

#### Risk factors and biochemical indicators

The researcher interviewed the patient to confirm the difference between the four risk factors of the research participants, namely obesity, smoking, excessive drinking more than eight times a month, and lack of exercise, before and after 12 weeks. The body mass index(BMI) was used to determine the degree of obesity, and the height and weight for this were measured using an automatic height scale (DS-103 M, Garden Zenix). The participants wore light attire and did not carry any belongings. Biochemical indicators were collected from electronic medical records, and blood test were performed after fasting for at least 8 h to measure fasting blood sugar, total cholesterol, high-density lipoprotein cholesterol, and triglyceride levels.

### Statistical analysis

The data was analyzed using SPSS WIN 22.0. Descriptive statistics, such as frequency, mean, and standard deviations, were obtained. Among subjects with risk factors, the Chi-square test and Fisher’s exact test were used to verify the difference in pre- and post-change changes between the experimental and control groups. A hypothetical test of the effectiveness of the risk factor-tailored education program was performed using the Chi-square test and independent t-test. Cronbach’s α coefficient was used to verify the reliability of the tool. All statistical significance levels were set at 0.05.

### Ethical considerations

Prior to data collection, ethical approval for this study was obtained from the institutional review board (Approval NO. SGPAIK 2021-02-012) of the Inje University Sanggye Paik hospital in Seoul, Korea. Data collection was carried out after the researcher obtained informed consent from the subjects who wished to voluntarily participate in this study. After explaining the purpose of the study and the benefits and disadvantages of participating in the study, it was explained that it was possible to withdraw consent during the study. Researcher explained to all participants that all data acquired would not be used for any purposes than research.

## Results

### Effectiveness of the risk factor-tailored patient education program

There were no significant differences in general and clinical characteristics, autonomous motivation, resilience, self-care compliance, and physical and biochemical indicators between the experimental and control groups (Table 2).

The autonomous motivation score of the experimental group increased by 0.37 points, from 4.96 points before the intervention to 5.33 points after the intervention. The change was significantly greater than that of the control group, which decreased from 4.75 points to 4.67 points (t = 4.46, *p* < .001). The resilience score of the experimental group increased by 0.28 points from 2.85 points before the intervention to 3.13 points after the intervention; this change was significantly greater than that of the control group, which decreased from 2.88 points to 2.85 points (t = 7.08, *p* = .003). The self-care implementation score of the experimental group increased by 0.27 points from 2.66 points before the intervention to 2.94 points after the intervention; this change was significantly greater than that of the control group, which decreased from 2.68 points to 2.66 points (t = 5.56, *p* < .001). There were no significant pre- to post-intervention changes in fasting blood sugar, total cholesterol, high-density lipoprotein cholesterol, and triglycerides (Table 3).

To investigate changes between pre and post-test, four risk factors (obesity, current smoking, heavy drinking more than 8 times a month, and lack of exercise) were identified, and there was a significant decrease in BMI in the experimental group (t=-4.92, p = .005). For the other three risk factors, the pre- and post-values of the risk factors were compared between the two groups, targeting only subjects with the risk factors. As a result of the Chi square test, current smoking significantly decreased in the experimental group from 19 subjects before the intervention to 4 subjects after the intervention, which was significantly difference (χ^2^ = 11.5, p = .001). However, there was no significant difference in excessive drinking and exercise between the two groups (Table 3).

**Table 2:** Homogeneity test of characteristics and dependent variables of subjects

Variables	Categories	Experimental(n = 32)	Control(n = 32)	χ^2^ or t	*p*
		n(%) or M ± SD	n(%) or M ± SD		
Age, yr		55.69 ± 6.46	57.25 ± 6.24	-0.98	0.523
Gender	Male	25(78.1)	26(81.3)	0.09	0.756
	Female	7(21.9)	6(18.8)		
Marital status	Married	22(68.8)	21(65.6)	0.71	0.790
	Unmarried/Divorced/Widowed	10(31.3)	11(34.4)		
Living with	Spouse/Children/Others	25(78.1)	22(68.8)	0.72	0.396
	Alone	7(21.9)	10(31.3)		
Education	≤ Middle school	9(28.1)	9(28.1)	2.25	0.522
	High school	11(34.4)	11(34.4)		
	≥ College	12(37.5)	12(37.5)		
Medical diagnosis	STEMI	9(28.1)	5(15.6)	1.60	0.448
	NSTEMI	14(43.8)	15(46.9)		
	Unstable angina	9(28.1)	12(37.5)		
Comobidities^†^	None	14(43.8)	8(25.0)	2.49	0.114
	Hypertension, yes	16(50.0)	23(71.9)	3.21	0.073
	Diabetes mellitus, yes	6(18.8)	9(28.1)	0.78	0.376
	Dyslipidemia, yes	6(18.8)	9(28.1)	0.78	0.376
	Cerebrovascular disease, yes	3(9.4)	2(6.3)	0.35	0.554
Smoking	Never/ Ex-smoker	13(40.6)	20(62.5)	3.12	0.209
	Current smoker	19(59.4)	12(37.5)		
Alcohol drinking	Heavy drinker(≥ 8 times/month)	8(25.0)	11(34.4)	1.16	0.558
Regular exercise	Never	12(37.5)	6(18.8)	3.69	0.157
Family CVD history	Yes	18(56.3)	11(34.4)	3.09	0.079
Body mass index	≥ 25 kg/m²	20(62.5)	18(56.3)	0.25	0.611
Fasting blood sugar	≥ 100 mg/dL	28(87.5)	26(81.3)	0.47	0.491
Total cholesterol	≥ 200 mg/dL	14(43.8)	8(25.0)	2.49	0.114
HDL-cholesterol	< 40 mg/dL	10(31.3)	9(28.1)	0.07	0.784
Triglyceride	≥ 150 mg/dL	25(78.1)	19(59.4)	2.61	0.106
Autonomous motivation		4.96 ± 1.32	4.75 ± 1.13	0.69	0.242
Resilience		2.85 ± 0.60	2.88 ± 0.46	-0.22	0.085
Self-care compliance		2.66 ± 0.62	2.68 ± 0.54	-0.15	0.692

M = mean; SD = standard deviation; STEMI = ST-segment elevation myocardial infarction; NSTEMI = Non ST-segment elevation myocardial infarction; HDL = high-density lipoprotein; CVD = cardiovascular disease; ^†^multiple responses

**Table 3:** Comparison of differences of autonomous motivation, resilience, self-care compliance, and physiological indicators between two groups

Variables	Group	Pre-test	Post-test	Difference (Post-Pre)	t/ χ^2^	*p*
		M ± SD, n(%)	M ± SD, n(%)			
Autonomous motivation	Experimental	4.96 ± 1.32	5.33 ± 1.06	0.37 ± 0.53	4.46	< 0.001
	Control	4.75 ± 1.13	4.67 ± 1.13	-0.07 ± 0.19		
Resilience	Experimental	2.85 ± 0.60	3.13 ± 0.47	0.28 ± 0.22	7.08	0.003
	Control	2.88 ± 0.46	2.85 ± 0.49	-0.02 ± 0.09		
Self-care compliance	Experimental	2.66 ± 0.62	2.94 ± 0.49	0.27 ± 0.30	5.56	< 0.001
	Control	2.68 ± 0.54	2.66 ± 0.53	-0.02 ± 0.05		
Fasting Blood Sugar(mg/dL)	Experimental	149.72 ± 52.26	120.25 ± 25.76	-29.46 ± 43.13	-1.30	0.112
	Control	136.41 ± 43.96	119.87 ± 28.93	-16.53 ± 35.94		
Total cholesterol(mg/dL)	Experimental	181.56 ± 50.43	130.93 ± 21.70	-50.62 ± 51.47	-1.77	0.500
	Control	165.75 ± 52.92	140.21 ± 38.73	-25.53 ± 61.25		
HDL-cholesterol(mg/dL)	Experimental	44.03 ± 7.60	44.68 ± 10.03	0.65 ± 8.33	-1.98	0.812
	Control	43.41 ± 9.64	48.34 ± 10.29	4.93 ± 8.87		
Triglyceride(mg/dL)	Experimental	207.91 ± 127.56	141.71 ± 72.89	-66.17 ± 115.12	-1.13	0.746
	Control	174.25 ± 107.64	139.96 ± 71.40	-34.28 ± 109.83		
Body mass index(kg/m²)	Experimental	26.54 ± 3.36	26.10 ± 3.08	-0.44 ± 0.60	-4.92	0.005
	Control	26.25 ± 3.18	26.41 ± 3.28	0.19 ± 0.40		
Lack of exercise(n = 49)^†^	Experimental	27(55.1)	26(54.2)			1.00*
	Control	22(44.9)	22(45.8)			
Current smoking (n = 31) ^†^	Experimental	19(61.3)	4(28.6)		11.5	0.001
	Control	12(38.7)	10(71.4)			
Heavy drinking(≥ 8times/month) (n = 12) ^†^	Experimental	6(18.8)	4(12.5)			0.567*
	Control	6(18.8)	3(9.4)			

The experimental and control groups are 32 each. M = mean; SD = standard deviation; HDL = high-density lipoprotein

^†^Analysis only on subjects with risk factors

*Using Fisher’s exact test

## Discussion

This study aimed to verify the effectiveness of individual education that focuses on the participant’s risk factors and emphasizes autonomy for improvement. As a result of confirming the degree of risk factor improvement through interview after 12 weeks, other risk factors were not improved, but the smoking rate was significantly reduced from 59 to 13% in the experimental group compared to the control group. This supports the results that one-on-one smoking cessation education for male hospitalized patients who underwent PCI in the experimental group significantly improved knowledge and attitudes toward smoking [26], and that the efficacy enhancement education program was effective in quitting smoking [27]. Smoking is the most important predictor of acute myocardial infarction in individuals aged < 65 years. According to the Korean registry data for acute myocardial infarction the smoking rate was 76.4% [28]. In addition, as a result of follow-up of smokers and non-smokers one year after PCI, continuing smoking significantly increased the mortality rate compared to quitting smoking [6], suggesting that smoking cessation education for middle-aged smokers after PCI is absolutely necessary.

As a result of verifying the effectiveness of the education program in this study, autonomous motivation was significantly improved in the experimental group compared with the control group. This aligns with the results of previous studies where autonomous motivation of patients who participated in a cardiac rehabilitation program was identified as a predictor of motor behavior [29]; the higher the autonomous motivation, the stronger the willingness to regulate and strive for activity, resulting in the continued maintenance of physical activity performance [30]. In addition, the study supports previous findings that autonomous motivation scores increased significantly after six weeks of providing motivational interviews and self-regulation coaching to patients with rheumatoid arthritis, in that the counseling and education programs promoted autonomous motivation [31]. In a study comparing a group of patients receiving follow-up care without recurrence for more than a year to determine the effect on recurrence after PCI, the group with low autonomy support had a 3.91-fold increased risk of recurrence of CAD and lower average score for self-care compliance compared with the group with higher autonomy support [12]. This result indicates the importance of autonomy and self-care compliance.

In the present study, resilience was significantly higher in the experimental group than in the control group after 12 weeks of intervention. There is a lack of prior research that has identified changes in resilience due to educational programs for patients who have received PCI. In a study of seniors who had high blood pressure using the senior welfare center, participants with experience receiving health education related to hypertension showed higher resilience than those who did not; resilience was identified as the most influential factor in self-care outcomes, similar to the results of this study [32].

One study identified the effectiveness of a 12-week coaching program for improving resilience in middle-aged women. It showed significant changes in self-regulation and interpersonal skills (sub-areas of post-interventional resilience) [14]. In another study, self-regulation and similar control showed significant differences before and after intervention, but there were no significant differences in supportive relationships similar to interpersonal skills. This is due to the difference in the resilience tools used in each study. Although the subjects of the previous study were middle-aged women in their 40 and 50 s, this study examined middle-aged men and women aged 40–64. Similar studies of people with CAD have shown that the higher the resilience, the higher the health-promoting behavior [13]; consistent with the results of our study.

Finally, the risk factor-tailored education significantly increased self-care compliance in the experimental group. This supports the findings of another study [33] which reported that patients with heart failure were less symptomatic and had better physical indicators (such as weight) post-intervention; it also found that patients with chronic disease risk factors may have improved self-care by recording their blood pressure and blood sugar levels to make them aware of their current condition. Prior studies on patients with coronary artery disease using education and telephone counseling interventions from admission to discharge have also reported improvements in self-care compliance [34]. Another study examined self-care compliance in outpatients who were diagnosed for the first time with CAD and received follow-up PCI; it found that 63% of the participants with low self-care compliance were less than 12 months after PCI [35], indicating that self-care compliance must be religiously promoted within the first year of receiving PCI.

The present study also explored differences in physical and biochemical indicators pre- and post-intervention. Among such indicators, only body mass index showed a significant decrease in the experimental group, compared with the control group. These results support the results of other studies showing significant differences in body mass index and waist circumference as a result of interventions focused on physical activity [36], and that long-term diet management and exercise can improve body mass index and abdominal obesity rates [37]. The interventions in this study provided tailored interventions for risk factors, such as obesity and lack of physical activity. However, this is multidisciplinary education to promote self-care compliance and will require interventions using objectively measurable variables. Moreover, the average fasting blood sugar decreased from 149 mg/dL to 120 mg/dL after the intervention. However, 120 mg/dL is not within normal limits, and the change was not significant. Among the study subjects, 18.8% of the patients who were diagnosed with chronic diseases, such as diabetes and dyslipidemia, and were constantly taking related medications were judged to have influenced their blood tests. In a systematic review of the effect of counseling on the lifestyle of patients with cardiovascular risk factors, the reduction in total cholesterol and triglyceride levels was most effective 12 to 24 months after the intervention [38]. Therefore, the 12-week intervention in this study has limitations in improving biochemical indicators, so a longer-term intervention is needed.

A limitation of this study is that though it was randomized, it did not employ a double-blind procedure; therefore, researchers may have involuntarily influenced the results of the study. In addition, participants were recruited from only one University Hospital in Korea restricting the generalizability of the results of the study to people with middle-aged coronary artery disease in Korea. Moreover, the use of self-reported questionnaires may have caused measurement errors.

Despite its limitations, this study is significant because it confirms that the program had positive outcomes for middle-aged patients with CAD who have a high prevalence of chronic diseases and need to engage in long-term self-care. Among other things, it is believed that the intervention method, nurse-led counseling and training, telephone consultations, and multimedia text message transmissions tailored to the risk factors were effective in increasing the autonomous motivation of the participants through productive interaction between nurses and patients. In particular, it is meaningful in that an educational program emphasizing the role of patients who have to live as a chronic patient for a long period of time after PCI was developed, and the effects on autonomous motivation, resilience, and self-care compliance were confirmed.

## Conclusions

This study confirmed that risk factor-tailored education by nurses for 12 weeks early after discharge to middle-aged patients who had undergone PCI significantly improved autonomous motivation, resilience, and self-care compliance. It was also effective in reducing current smoking rate and body mass index among risk factors. This study suggests that in order to increase self-care for middle-aged patients who still have a long life expectancy, nurses should provide education at the beginning of discharge to emphasize autonomy as a chronic disease patient, foster resilience, and recognize risk factors. Through future research, it is necessary to establish the role of dedicated nurses who continuously evaluate and manage the level of self-care and risk factor improvement in middle-aged patients with cardiovascular disease and to verify the long-term effects.

### Electronic supplementary material

Below is the link to the electronic supplementary material.

**Supplementary Material 1:** Interview manual

**Supplementary Material 2:** CONSORT 2010 checklist

## Data Availability

Not applicable.

## Abbreviations

CAD: Coronary Artery Disease
PCI: Percutaneous Coronary Intervention
