# Supplementary material for: Effect of risk factor-tailored autonomy enhancement education in the first-time middle-aged patients undergoing percutaneous coronary intervention: a randomized controlled trial
Source: BMC Nurs. 2023 Dec 18;22:479. doi: 10.1186/s12912-023-01654-6 (PMC10726554; doi:10.1186/s12912-023-01654-6)
Supplement: Supplementary file 1 — Supplementary Material 1: Interview manual [file 12912_2023_1654_MOESM1_ESM.pdf]

Interview Manual

Research Title: Effect of Risk Factor-tailored Autonomy Enhancement Education in the First-time Middle-aged Patients undergoing Coronary Intervention

---

Hello? The following is a description of a study to develop a patient education program tailored to risk factors for middle-aged patients who underwent coronary intervention and verify its effectiveness. After reviewing this document, if you wish to participate in the study, please sign the consent form.

---

1. Study purpose

The purpose of this study was to improve the clinical prognosis of middle-aged patients who underwent coronary artery intervention. This is to verify the effect after applying the customized smart patient education program.

2. Study participants

Subjects eligible to participate in this study were those in their 40s who were first diagnosed with coronary artery disease and underwent interventional surgery. Middle-aged patients between the ages of 69 and older who have one or more risk factors for coronary artery disease. In addition, there is no communication barrier and it is possible to understand the purpose of the study and write consent as it is possible to read Korean., and the total number of study subjects is 70, 35 in the experimental group and 35 in the control group.

3. How to conduct research

In this study, subjects who underwent coronary artery intervention and met the selection criteria were enrolled in the Microsoft Excel program. Select the experimental group and the control group using Ram's random function. The experimental group and the control group respectively. Since the assignment is 1:1, subjects participating in the study have a 50% chance of being assigned to the experimental group or the control group. When assigned to the experimental group, customized patient education program based on individual risk factors will be provided individually to strengthen self-motivation and autonomy. After coronary artery intervention, interviews and education with the researcher are conducted for about 50 minutes before discharge. The contents of the interview are the risk factors that the subject is aware of and what kind of education and counseling will be done in the future. Interviews are conducted in consideration of autonomy, such as whether or not the interview was conducted and the individuality of the applicant. On the day of the outpatient visit after two weeks of education on specific risk factors, 1:1 education will be conducted for about 50 minutes. Until the 11th week, subjects participate voluntarily, during which a total of 2 phone interviews will be conducted, during which the researcher will identify the patient's barriers. After completing the 12-week educational program, a final follow-up survey will be conducted at the outpatient visit 12 weeks later. In the case of the control group, routine education about diseases and procedures is conducted before discharge after coronary intervention.

Your general characteristics, disease-related characteristics, body parameters, and biochemical characteristics can be checked through your hospital medical records, and responses to a questionnaire measuring the following three variables will be collected. Thank you so much for your assistance. Researchers.
